# Evaluation of MAGNet, a long-lasting insecticidal mosquito net against *Anopheles fluviatilis* in experimental huts in India

**DOI:** 10.1186/s12936-019-2692-3

**Published:** 2019-03-06

**Authors:** Gunasekaran Kasinathan, Sudhansu Sekhar Sahu, Vijayakumar Tharmalingam, Subramanian Swaminathan, Manju Rahi, Jambulingam Purushothaman

**Affiliations:** 1 ICMR-Vector Control Research Centre, Medical Complex, Indira Nagar, Puducherry, 605006 India; 2 Indian Council of Medical Research, New Delhi, India

**Keywords:** Alpha-cypermethrin, *Anopheles fluviatilis*, Experimental huts, MAGNet, India

## Abstract

**Background:**

MAGNet LN is a wash resistant long-lasting insecticidal (polyethylene) net (LLIN) in which the alpha-cypermethrin insecticide is incorporated within the 150 denier high density polyethylene monofilaments of the nets. The bio-efficacy of MAGNet LN was reported to be high even after 25 washes. The LN met the WHO criteria of Phase I evaluation and obtained recommendation from the World Health Organization Pesticide Evaluation Scheme (WHOPES) for Phase II trial. For registration of the LN in India, the current study was conducted to evaluate its efficacy after 20 or 25 washes compared to negative control (untreated net) and positive control (Duranet LN) in experimental huts against a wild, free flying pyrethroid susceptible population of *Anopheles fluviatilis* in terms of deterrence, blood-feeding inhibition, mortality and induced exophily.

**Methods:**

The evaluation was carried out in six experimental huts located at Kandhaguda village in Malkangiri district, Odisha state following the WHO guidelines.

**Results:**

The study showed that 25 times washed MAGNet LN produced 100% mortality in cone bioassays before and after hut evaluation. MAGNet washed 25 times did not differ significantly from all other treated nets in terms of deterring hut entry, induced exophily, blood feeding inhibition and causing mortality of *An. fluviatilis.*

**Conclusions:**

MAGNet LN showed extended wash resistance retaining its bio-efficacy up to 25 washes and met the WHOPES requirement of passing Phase II evaluation.

**Electronic supplementary material:**

The online version of this article (10.1186/s12936-019-2692-3) contains supplementary material, which is available to authorized users.

## Background

Malaria remains one of the major challenges and an important cause of morbidity and mortality in India today [1]. India contributes two third (n = 1,087,285) of the total malaria cases in South East Asia during 2016 [2]. The malaria burden in the country has been sustained by the presence of efficient vectors that maintain high levels of transmission [3]. Vector control remains an essential component and a fundamental element of malaria control strategy in India [4]. Indoor residual spraying (IRS) and insecticide-treated nets (ITNs) are the two frontline and mostly applicable interventions used since last two decades for the control of malaria vectors in the country [5]. The challenge for the success of these tools is to ensure that these interventions should reach a major proportion of the population through universal coverage [6]. Due to the hurdles of achieving high coverage and quality, IRS was replaced with ITNs in most of the areas during 1990′s and has been accepted as a touchstone for malaria vector control [7]. ITNs were well received since last three decades and have been proven to give protection against malaria in many countries including India [3, 6]. However, successful implementation of ITNs was hampered by several technical, operational, economic and social factors [5]. Retrieval of nets from the households after distribution for retreatment was the main operational challenge in large-scale implementation of ITNs [7].

To overcome this problem, use of long-lasting insecticidal nets (LLINs) has been encouraged by the World Health Organization (WHO) and extended to hard core malarious areas since last one decade [5]. In most of the LLINs made in the factory, insecticide is either incorporated into the fibres or coated on the fibres [3]. LLINs usually retain insecticidal bio-efficacy for 3 years without re-treatment and withstand 20 washes [8]. At present, only a few LNs (LN indicates a particular brand as approved by the WHO), which claim to withstand more than 20 washes have been put under different phases of evaluation [9].

MAGNet is one such wash resistant new LN in which the alpha-cypermethrin insecticide is incorporated within the 150 denier high density polyethylene (HDPE) filaments which diffuses to the surface slowly (controlled release of insecticide) and a small amount of insecticide available on the surface (bio-availability) is sufficient enough to kill the mosquitoes [9]. Washing the net removes the insecticide on the surface. But, the advantage of this LN is the immediate replenishment of insecticide within the filaments after washing [9]. Another advantage of MAGNet is that the formulation used in the LN restores the bio-efficacy within 24 h and heating is not required to accelerate restoration of bio-efficacy after washing. The bio-efficacy of MAGNet was reported to be high even after 25 washes [10]. This LN has met the WHO criteria of Phase I study by causing a mosquito knock down (KD) ≥ 95% and/or mortality ≥ 80% for 20 washes and obtained recommendation from the WHOPES for a Phase II trial [9]. Further, the LN has obtained national (India) clearance for Phase II evaluation which is required for the product to be registered for its use in the country. Therefore, the current study was carried out to evaluate the efficacy of MAGNet LN washed 20 or 25 times compared to the reference LN, Duranet, washed 20 times in experimental huts (Phase II evaluation) against the natural pyrethroid susceptible population of *Anopheles fluviatilis* in terms of deterrence, blood-feeding inhibition, mortality and induced exophily.

## Methods

### Study area

The trial was conducted in the six experimental huts constructed in Kandhaguda village of Malkangiri District (latitude 18°25′N and longitude 81°58′E), the southernmost part of Odisha state. This study area is hilly and forested with many seasonal and perennial streams and rivers. *Anopheles fluviatilis* is the predominant malaria vector in this area and is abundant during September–February (peaks in November to December) [1]. *Anopheles culicifacies* is the second vector, abundant during March–August (peak in August) [1]. The district is hyper endemic for malaria with annual parasite incidence (API) ranged from 18.5 to 60.3 with 30 recorded deaths during the last 5 years (2013–2017) (Source: CDMO Office, Malkangiri). More than 90% of the total malaria cases are due to *Plasmodium falciparum* infection [11].

### Design of experimental huts, experiment arms and net washing

The specifications of the experimental huts, the process of net washing, cone bioassays and chemical analysis have been described elsewhere [5]. Washing of nets was done at Indian Council of Medical Research-Vector Control Research centre (ICMR-VCRC), Puducherry following the WHO washing protocol [12]. The nets for the trial (MAGNets, Duranets and untreated polyester nets) were provided by V.K.A. Polymers, Karur, Tamil Nadu. The trial had six comparison arms viz., unwashed MAGNet, MAGNet washed 20 times, unwashed Duranet (positive control), Duranet washed 20 times (positive control), polyester untreated net (negative control) and MAGNet washed 25 times.

### Cone bioassays and chemical analysis

Prior to any wash and after 20 or 25 washes, the two additional nets were used for cone bioassays and chemical analysis. Bioassays and chemical analysis were performed on adjacent pieces of the same nets. WHO-prescribed cone bioassays were done with laboratory reared susceptible fully-fed *Anopheles stephensi* before any wash and after 20 or 25 washes at ICMR-VCRC, Puducherry.

After washing and bioassays, all nets for the trial were transported to the field site for evaluation in experimental huts. Prior to hut-evaluation, bioassays were performed using wild caught pyrethroid-susceptible *An. fluviatilis* on one net randomly selected from the six replicate nets of each arm. At the end of hut-evaluation, as adequate number of *An. fluviatilis* could not be obtained in the study site, bioassays were done exposing wild caught pyrethroid-susceptible *Anopheles jeyporiensis* on one used net randomly selected from each arm.

For chemical analysis, 5 pieces were taken from one of the two additional nets of each of the six arms prior to any wash and after 20 or25 washes following the WHO guidelines [12]. At the end of the trial, one used net from each of the six arms was sampled in the same way as described above. The net samples were labelled and packed in aluminium foil and sent to Walloon Agricultural Research Centre, CRA-W, Gembloux, Belgium for insecticide content analysis.

### Preparation of nets

Six holes (each hole with 4 cm × 4 cm) per net were made deliberately in all nets of the six arms in order to simulate the conditions of a torn net and to put emphasis on testing whether the insecticide treatment, rather than the net, effectively prevents mosquito biting on sleepers [12].

### Ethical clearance

Clearance was obtained from the ICMR-VCRC Human Ethics Committee to involve twelve adult volunteers in the hut evaluation. For participating in the study, written informed consent was obtained from all the volunteers and they were informed about the study procedure. During the entire study period, all volunteers were monitored for fever or any adverse effects due to the use of LLINs.

### Selection of volunteers for hut evaluation, acclimatization and hut suitability

The procedure of selection of volunteers, the process of acclimatization and the assessment of hut suitability have been described elsewhere [5].

### Experimental hut evaluation

After ensuring hut suitability, evaluation of nets was commenced in six experimental huts from 09.11.2015 to 12.03.2016 (18 weeks). Since, adequate number of the vector species could not be collected in untreated arm during this period, the evaluation was continued for another 18 weeks from 07.11.2016 to 11.03.2017. Each arm comprised of six nets and one net from each arm was tested for each night per week. On the previous day of mosquito collection, in the evening, huts were cleaned and ant gel paste was placed in two corners of the hut and verandah to avoid scavenging. Clean white cloths were spread on the floor inside the hut and the verandah, and the gutter was filled with water. In each hut, the allotted treatment net was tied inside the hut and bedding materials were placed. The volunteers entered the experimental huts at about 19.00 h and slept under the nets assigned to that hut and remained inside until 05.30 h in the morning.

### Rotation of experiment arms and sleepers and mosquito collection and processing

The nets and the volunteers were rotated according to Latin square design. The schemes of rotation, mosquito collections and processing have been described elsewhere [5].

### Statistical analysis

The data were analysed to estimate the effect of the six arms in terms of deterrence, induced exophily, blood-feeding inhibition, and total mortality. The number of *An. fluviatilis* caught in each hut was recorded by day and checked for variance and mean. Logistic regression was used for proportional data and negative binomial regression was used for numeric data; adjustments were made for the effect of hut and sleeper (Stata software, Version 10). For overall comparison, untreated net (negative control) was kept as reference category. Further comparison between the treatment arms was made from the 95% confidence intervals of the incidence rate ratio (IRR) or odds ratios, as applicable. The mean per-hut density (PHD) of *An. fluviatilis* was compared between experimental huts and village huts using one way ANOVA. The number of occasions that recorded entry of the vector species into the experimental huts was compared between different arms using χ^2^ test. A ‘p’ value < 0.05 was considered as statistically significant.

## Results

### Species composition

A total of 216 collections were made from the experimental huts for each of the six experiment arms. In total, 1808 mosquitoes were collected and among them, *An. fluviatilis* formed 19.0%, *An*. *culicifacies* 15.7%, other anophelines 31.4%, and 33.8% was culicines. Since, the objective of the study was to evaluate the efficacy of MAGNet LN against the pyrethroid susceptible vector, *An. fluviatilis*, detailed analysis of data was carried out for this vector species only. However, the data collected for pyrethroid resistant *An*. *culicifacies,* the other vector species, present in the study area, are provided in Additional file 1.

### Hut entry

The results of hut entry of *An. fluviatilis* for the six arms are presented in Table 1. With untreated net, there was no hut entry of *An. fluviatilis* on 102 occasions (out of 216 occasions/collections), whereas, in the treated arms: unwashed Duranet, Duranet washed 20 times, unwashed MAGNet, MAGNet washed 20 times and MAGNet washed 25 times, zero entry was observed on 201, 192, 194, 189 and 189 occasions, respectively signifying a marked deterrent effect of alpha-cypermethrin in the treated nets. The number of occasions of zero entry in the treated arms was significantly greater than the untreated arm (negative control) (p < 0.05 by χ^2^ test), but not significantly different from the positive control (unwashed Duranet) (p > 0.05 by χ^2^ test). While, 216 *An. fluviatilis* were collected from the untreated arm, only 128 were obtained from all the five treated arms (ranged from 16 to 30) from the 216 collections in each arm. The deterrent effect of the synthetic pyrethroid (SP) was further evidenced from the collection of seven *An. fluviatilis* inside the untreated net compared to none under the treated nets in spite of the presence of holes.

**Table 1:** Hut entry of *Anopheles fluviatilis* in treated and untreated arms

Experiment arms	Number of collections	Number entered (entry)^a^	Range	Mean entry ± SE	Incidence rate ratio (IRR)	95% CI	p
Untreated net (negative control)	216	216	0–7	1.0 ± 0.09	1.00^b^	–	–
Unwashed Duranet (positive control)	216	16	0–2	0.07 ± 0.02	0.074	0.043–0.127	0.000
Duranet washed 20 times (positive control)	216	27	0–2	0.13 ± 0.03	0.125	0.081–0.193	0.000
Unwashed MAGNet	216	30	0–8	0.14 ± 0.04	0.139	0.091–0.211	0.000
MAGNet washed 20 times	216	27	0–1	0.13 ± 0.02	0.125	0.081–0.193	0.000
MAGNet washed 25 times	216	28	0–2	0.13 ± 0.02	0.129	0.084–0.199	0.000

^a^From 216 collections in each arm; *SE* Standard error

^b^Reference category: untreated polyethylene net (negative control)

Negative binomial regression analysis revealed an over-dispersion of the hut entry (non-random) of *An. fluviatilis* [alpha that measures over-dispersion = 0.82 (95% CI 0.52–1.30), χ^2^ = 46.5, df = 5, p < 0.05] and justified the analysis using negative binomial regression as well. Overall, hut entry of the vector species differed significantly between the six experiment arms (χ^2^ = 230.16, df = 5, p < 0.05). Compared to the untreated arm, the hut entry was significantly reduced in all the treated arms (p < 0.05). Among the treated arms, the entry was the lowest with unwashed Duranet as observed from the IRR. However, the 95% CI for the IRR showed no significant difference in entry between the five treated arms indicating their comparable deterrent effect (Table 1) (Additional file 2).

### Exit (induced exophily)

The exit rate from the huts with unwashed Duranet, Duranet washed 20 times, unwashed MAGNet, MAGNet washed 20 times and MAGNet washed 25 times ranged from 77.8 to 96.7% and in the hut with untreated net, the exit rate was 51.4%. The day-wise collections of exited mosquitoes in each arm over the evaluation period of 36 weeks were subjected to logistic regression analysis by taking exit of mosquitoes as dependent variable and the six experiment arms as categorical covariates, keeping the untreated net (negative control) as the reference category. Overall, there was a significant difference in exit rate between the six experiment arms (χ^2^ = 54.34, df = 5, p < 0.05). Compared to the negative control, all the five treated arms induced significantly a greater exophily (p < 0.05). Among the treated arms, unwashed MAGNet (96.7%) followed by unwashed Duranet (93.8%) induced relatively higher exophily than the washed nets of these two arms. However, 95% CI for the odds ratios showed no significant difference between the treated arms (Table 2). The treatment arms of the candidate LN were not significantly (χ^2^ = 6.49, df = 4, p = 0.16) different from positive control in terms of inducing exophily (Additional file 2).

**Table 2:** Exit of *Anopheles fluviatilis* from the huts with treated and untreated arms

Experiment arms	Number of collections	Number entered^a^	Number exited	Exit rate (%)	odds ratio	95% CI	p
Untreated net (negative control)	216	216	111	51.4	1.00^b^	–	–
Unwashed Duranet (positive control)	216	16	15	93.8	14.189	1.842–109.315	0.011
Duranet washed 20 times (positive control)	216	27	23	85.2	5.439	1.819–16.255	0.002
Unwashed MAGNet	216	30	29	96.7	27.432	3.671–204.992	0.001
MAGNet washed 20 times	216	27	21	77.8	3.311	1.286–8.524	0.013
MAGNet washed 25 times	216	28	23	82.1	4.351	1.596–11.867	0.004

^a^From 216 collections in each arm

^b^Reference category: untreated polyethylene net (negative control)

### Blood feeding rate

In the huts, with untreated nets, the blood feeding rate was 81.9%. Among the five treatment arms, the feeding rate was the lowest (highest blood feeding inhibition) with unwashed MAGNet (43.3%) followed by MAGNet washed 20 times (48.1%). None of the treated arms prevented blood feeding completely, as the feeding rate varied between 43.3 and 68.8% (Table 3). Logistic regression analysis showed that overall, the feeding rate differed significantly among the six experiment arms (χ^2^ = 31.01, df = 5, p < 0.05) While untreated polyethylene net was taken as reference category (negative control), the feeding rate was significantly lower with all the treated arms except unwashed Duranet (p = 0.20) (Table 3). However, between the five treatment arms, the feeding rate did not differ significantly as shown by the 95% CI for the odds ratios, as shown in Table 3 and Additional file 2.

**Table 3:** Blood feeding rate of *Anopheles fluviatilis* in treated and untreated arms

Experiment arms	No. of collections	Number entered^a^	Number fed	% fed	Odds ratio	95% CI	p
Untreated net (Negative control)	216	216	177	81.9	1.00^b^	–	–
Unwashed Duranet (Positive control)	216	16	11	68.8	0.485	0.159–1.475	0.202
Duranet washed 20 times (Positive control)	216	27	17	63.0	0.375	0.159–0.880	0.024
Unwashed MAGNet	216	30	13	43.3	0.168	0.076–0.375	0.000
MAGNet washed 20 times	216	27	13	48.1	0.205	0.089–0.469	0.000
MAGNet washed 25 times	216	28	18	64.3	0.397	0.169–0.925	0.032

^a^From 216 collections in each arm

^b^Reference category: untreated polyethylene net (negative control)

### Mortality

The immediate mortality was zero in all the treated arms throughout the hut trial, except unwashed MAGNet, that caused an immediate mortality of 3.3%. The total mortality (immediate + delayed) with unwashed Duranet, Duranet washed 20 times, unwashed MAGNet, MAGNet washed 20 times and MAGNet washed 25 times was 50.0%, 44.4%, 56.7%, 48.1% and 35.7%, respectively (Table 4). Since, mortality was zero with the negative control, this arm was excluded from the grouped data and the positive control was used as reference category for logistic regression analysis to compare the efficacy of the five treated arms. Overall, there was no significant difference in total mortality among the treated arms (χ^2^ = 2.72, df = 4, p = 0.61) (Table 4).

**Table 4:** Mortality of *Anopheles fluviatilis* in treated and untreated arms

Experiment arms	Number entered^a^	Immediate mortality (%)	Delayed mortality (%)	Total mortality (%)	Odds ratio	95% CI	p
Untreated net (Negative control)	216	0 (0)	0 (0)	0 (0)	–	–	–
Unwashed Duranet (Positive control)	16	0 (0)	8 (50.0)	8 (50.0)	1.0^b^	–	–
Duranet washed 20 times (Positive control)	27	0 (0)	12 (44.4)	12 (44.4)	0.800	0.232–2.763	0.724
Unwashed MAGNet	30	1 (3.3)	16 (53.3)	17 (56.7)	1.308	0.387–4.417	0.666
MAGNet washed 20 times	27	0 (0)	13 (48.1)	13 (48.1)	0.929	0.269–3.199	0.907
MAGNet washed 25 times	28	0 (0)	10 (35.7)	10 (35.7)	0.556	0.159–1.936	0.356

^a^From 216 collections in each arm

^b^Since, no mortality was recorded with untreated net (negative control), it was removed from the grouped data. Instead, the positive control, unwashed Duranet, was used as reference category for logistic regression analysis

### Residual action of the insecticide on the nets

Prior to any wash and after 20 washes, both MAGNet and Duranet caused 100% mortality (in cone-bioassays) of *An. stephensi,* susceptible to alpha-cypermethrin, while its mortality was zero against the untreated net. Even after 25 washes, MAGNet caused 100% mortality of *An. stephensi.* Just before experimental hut evaluation, bioassays were done with wild caught *An. fluviatilis* on both MAGNet and Duranet; which also showed 100% mortality. After the hut trial, bioassays were performed with the susceptible *An. jeyporiensis* and its mortality was 100% on both the LLINs (Table 5).

**Table 5:** Results of cone-bioassays^a^

Experiment arms	Before any wash		After 20 washes		After 25 washes		Prior to hut evaluation^a^		After hut evaluation	
	NE	CM (%)	NE	CM (%)	NE	CM (%)	NE	CM (%)	NE	CM (%)
Untreated net (Negative control)	50	0	50	0	–	–	50	0	50	0
Unwashed Duranet (Positive control)	50	100	50	100	–	–	50	100	50	100
Duranet washed 20 times (Positive control)	50	100	50	100	–	–	50	100	50	100
Unwashed MAGNet	50	100	50	100	–	–	50	100	50	100
MAGNet washed 20 times	50	100	50	100	–	–	50	100	50	100
MAGNet washed 25 times	50	100	50	100	50	100	50	100	50	100

*NE* number exposed, *CM* corrected mortality

^a^For cone-bioassays, *An. fluviatilis* was used prior to experiment hut evaluation and *An. jeyporiensis* was used after hut evaluation, whereas before any wash and after washes *An. stephensi* was used

### Chemical analysis

The mean ± SE alpha-cypermethrin content in three unwashed MAGNet was 5.8 ± 0.05, 5.6 ± 0.09 and 5.7 ± 0.09 g/kg, which complied with the target dose of 5.8 g/kg ± 25% for 100 denier yarn [4.4–7.3 g/kg]. The within-net variation of alpha-cypermethrin content which is expressed as relative standard deviation (RSD) on 5 different net pieces obtained from three unwashed nets was 1.7%, 3.2% and 3.3%, respectively, showing an acceptable homogeneity of the distribution of the active ingredient within the nets. After 20 or 25 washes, alpha-cypermethrin content was 5.3 ± 0.07 g/kg and 5.1 ± 0.05 g/kg, corresponding to overall insecticide retention of 95% and 90%, respectively. After the hut trial, alpha-cypermethrin content did not decrease a lot, as it was 5.2 ± 0.09, 5.0 ± 0.05 and 4.9 ± 0.04 g/kg in the MAGNet unwashed and washed 20 times or 25 times, respectively (Additional file 3).

The mean ± SE alpha-cypermethrin content in two unwashed Duranet was 7.1 ± 0.07 and 7.3 ± 0.02 g/kg. The nets complied with the target dose of 5.8 g/kg ± 25% for 100 denier yarn [4.4–7.3 g/kg]. The within-net variation (RSD) of alpha-cypermethrin content found on five different pieces cut from each of the two unwashed nets was 2.2% and 1.0%, showing an acceptable homogeneity of distribution of the active ingredient within the nets. After 20 washes, the alpha-cypermethrin content was 6.8 ± 0.04 g/kg with 93% retention of alpha-cypermethrin. After the hut evaluation, there was no significant decrease in the insecticide content, as it was 5.2 ± 0.14 and 6.1 ± 0.07 g/kg for the Duranet unwashed and washed 20 times, respectively. The alpha-cypermethrin content in the untreated net was lower the limit of quantification (< 0.05 g/kg) prior to any wash and after experimental hut study (Additional file 3).

## Discussion

Insecticide-treated bed nets offer protection from mosquito bites, thereby preventing the transmission of mosquito borne diseases [13]. Frequent washing of ITNs may lead to insecticide loss from the netting making them ineffective before due time. LLINs provide longer time protection against mosquito bites as they are wash resistant. Currently, there are many LNs used in malaria control programme, which have been recommended by WHOPES. Before receiving recommendation as LNs, the nets were shown to retain their effectiveness after undergoing 20 standardised washes [14]. Duranet, one such WHO recommended LN, which can resist up to 20 washes, was kept as positive control (reference net) in this trial, as this LN was similar to MAGNet LN in terms of insecticide, treatment technique and netting material [9]. The results of the experimental hut evaluation of MAGNet LN carried out in Odisha state, India against *An. fluviatilis*, an efficient malaria vector and susceptible to synthetic pyrethroids [1] are presented in this paper. The summarized results indicate that both MAGNet LN and Duranet LN caused 100% mortality of *An. stephensi* in cone-bioassays before any wash and after 20 (both MAGNet and Duranet) or 25 washes (only MAGNet LN). The bioassays on these LNs with *An. fluviatilis* prior to and with *An. jeyporiensis,* a pyrethroid susceptible local malaria vector [15] after hut evaluation also showed 100% mortality with all the treated arms. It is to be noted that MAGNet LN produced 100% mortality in cone bioassays even after 25 washes.

The MAGNet LN (washed or unwashed) produced significantly higher deterrent effect compared to untreated net. *Anopheles fluviatilis,* which was reported to be endophilic in the study area [1], exhibited endophily as well as exophily during the trial as evident from the 51.4% exit rate with untreated net, and the higher exit rate with the treated arms could be due to the induced exophily by the treatment. When unwashed MAGNet LN and MAGNet LN washed 25 times were compared, there was no significant difference in exit rate indicating an extended wash resistance of MAGNet LN. Further, the MAGNet LN washed 25 times inhibited blood feeding and caused mortality to a comparable level with the other treated arms, confirming its extended washed resistance.

The alpha-cypermethrin retention was 95% after 20 washes and 90% after 25 washes, demonstrating the bio-availability of the insecticide after washes. At the end of the study, the alpha-cypermethrin content among MAGNet unwashed, washed 20 times or 25 times did not vary much (5.2, 5.0 and 4.9 g/kg respectively) indicating that the MAGNet LN retained ≥ 90% active ingredient (alpha-cypermethrin) up to 25 washes and caused 100% mortality of the pyrethroid susceptible mosquitoes. A recent field evaluation of PermaNet 2.0 in the same study area showed that the annual washing rate per net varied from 3.2 to 6.6 [6]. Since, MAGNet LN withstands 25 washes and performed equal to the unwashed or 20 times washed nets, MAGNet LN would be well accepted by the community.

The revised WHOPES guidelines (2013) for testing LNs suggest to include a WHOPES-recommended LN with similar specifications to the candidate LN in terms of insecticide, treatment technique, netting material, and washing frequency (0 and 20 times) as a positive control [12]. Accordingly, Duranet LN was included in the current study as a positive control to ensure the equivalence or superiority of the candidate LN to the positive control. The results of the present study indicated that the performance of unwashed or washed (20 or 25 times) MAGNet LN was significantly higher than the untreated net (negative control) and comparable to/marginally better than the positive control (Duranet) in terms of mortality, deterrence, blood-feeding inhibition and induced exophily of the malaria vector, *An. fluviatilis* in experimental huts and thus the MAGNet LN fulfilled the WHOPES criteria of a long-lasting insecticidal net [12]. In India, phase II (experimental hut) evaluation of LNs is conducted only by ICMR-VCRC, as experimental hut facility is available only at this centre. Therefore, the findings of the current study were compared with the results of an earlier phase II study that tested Duranet and Interceptor LNs (alpha-cypermethrin treated nets) washed 20 times in the same site [5]. The comparison showed that MAGNet LN washed 20 times performed marginally better than Duranet and Interceptor LNs washed 20 times in terms of deterrence, induced exophily, blood feeding inhibition and mortality besides an extended wash resistance. Similar findings were observed in case of 25 times washed Interceptor LN, which met the performance standards for LNs with high efficacy [16]. Moreover, phase II studies of LNs are very limited not only in India and also in other countries. An experimental hut study conducted in north eastern Tanzania showed that Interceptor LN killed 92% of pyrethroid susceptible female *Anopheles gambiae* when unwashed and 76% when washed 20 times [17]. The results are in agreement with the findings of the current study.

One of the limitations of the current study was that adequate number of the vector species could not be collected during the first 18 weeks (November 2015–March 2016) and hence, evaluation was continued for another 18 weeks after a gap of 8 months (November 2016–March 2017). Another limitation was that due to the non-availability of *An. fluviatilis* in adequate number in the field, *An. stephensi* and *An. jeyporiensis,* which are not the target vectors in the study area, were used for bio-assays.

## Conclusions

The study concluded that MAGNet LN retained the bio-efficacy up to 25 washes. The efficacy of MAGNet LN, as observed during this trial fulfils the requirement set by WHOPES for phase II evaluation. In 2016, India initiated malaria elimination programme (2016–2030) at national level and emphasized mass campaign with LLIN intervention in endemic states of the country including Odisha for control of malaria vectors. During 2017, LLINs of Duranet LN were distributed in mass in all the endemic areas of the country. Since MAGNet LN performed with high deterrent effect and it had added benefit of retaining its bio-efficacy up to 25 washes, MAGNet LN could be a potential tool for use in the national malaria vector control programme.

## Additional files

**Additional file 1.** Entry, exit, blood feeding and mortality rate of *Anopheles culicifacies* in treated and untreated arms.

**Additional file 2.** Negative binomial regression analysis of entry, exit, feeding rate of *Anopheles fluviatilis,* using positive control net as reference category.

**Additional file 3.** Alpha-cypermethrin content in net samples before & after washing and after experimental hut trial.

## Abbreviations

LLIN: long-lasting insecticidal net
WHO: World Health Organization
WHOPES: World Health Organization Pesticide Evaluation Scheme
LN: long-lasting insecticidal net (when brand name is used)
IRS: indoor residual spraying
ITN: insecticide-treated bed net
HDPE: high density polyethylene
KD: knock down
API: annual parasite incidence
CDMO: Chief District Medical Office
CRA-W: Walloon Agricultural Research Centre
IRR: incidence rate ratio
PHD: per-hut density
ANOVA: analysis of variance
SP: synthetic pyrethroid
df: degrees of freedom
CI: confidence intervals
SE: standard error
RSD: relative standard deviation
