# Supplementary material for: Evaluation of MAGNet, a long-lasting insecticidal mosquito net against Anopheles fluviatilis in experimental huts in India
Source: Malar J. 2019 Mar 6;18:59. doi: 10.1186/s12936-019-2692-3 (PMC6404338; doi:10.1186/s12936-019-2692-3)
Supplement: Supplementary file 1 — Additional file 1. Entry, exit, blood feeding and mortality rate of Anopheles culicifacies in treated and untreated arms. [file 12936_2019_2692_MOESM1_ESM.docx]

**Additional file 1. Entry, exit and blood feeding and mortality rate of *An. culicifacies* in treated and untreated arms**

| **Experiment arms** | **Number of collections** | **Number entered** | **Number exited (%)** | **Number fed (%)** | **Total morality (%)** |
| --- | --- | --- | --- | --- | --- |
| Untreated net (Negative control) | 216 | 56 | 30 (53.6) | 41 (73.2) | 1(1.8) |
| Unwashed Duranet (Positive control) | 216 | 45 | 35 (77.8) | 19 (42.2) | 8 (17.8) |
| Duranet washed 20 times (Positive control) | 216 | 42 | 35 (83.3) | 17 (40.5) | 11 (26.2) |
| Unwashed MAGNet | 216 | 39 | 31 (79.5) | 19 (48.7) | 13 (33.3) |
| MAGNet washed 20 times | 216 | 42 | 30 (71.4) | 17 (40.5) | 14 (33.3) |
| MAGNet washed 25 times | 216 | 60 | 52 (86.7) | 30 (50.0) | 11(18.3) |
