# Supplementary material for: Evaluation of MAGNet, a long-lasting insecticidal mosquito net against Anopheles fluviatilis in experimental huts in India
Source: Malar J. 2019 Mar 6;18:59. doi: 10.1186/s12936-019-2692-3 (PMC6404338; doi:10.1186/s12936-019-2692-3)
Supplement: Supplementary file 2 — Additional file 2. Negative binomial regression analysis of entry, exit, feeding rate of Anopheles fluviatilis, using positive control net as reference category. [file 12936_2019_2692_MOESM2_ESM.docx]

| **Experiment arms** | **Entry** | | | **Exit** | | | **Feeding rate** | | |
| --- | --- | --- | --- | --- | --- | --- | --- | --- | --- |
|  | **Incidence rate ratio (IRR)** | **95% CI** | **P** | **Incidence rate ratio (IRR)** | **95% CI** | **P** | **Incidence rate ratio (IRR)** | **95% CI** | **P** |
| Unwashed Duranet (Positive control) | 1.0^@^ | - | - | 1.00*^@^* | - | - | 1.0^@^ | - | - |
| Duranet washed 20 times (Positive control) | 1.688 | 0.878-3.242 | 0.116 | 0.383 | 0.039-3.769 | 0.411 | 0.773 | 0.208-2.876 | 0.701 |
| Unwashed MAGNet | 1.875 | 0.987-3.563 | 0.055 | 1.933 | 0.113-33.125 | 0.649 | 0.348 | 0.097-1.250 | 0.106 |
| MAGNet washed 20 times | 1.688 | 0.878-3.242 | 0.116 | 0.233 | 0.025-2.145 | 0.199 | 0.422 | 0.115-1.547 | 0.193 |
| MAGNet washed 25 times | 1.750 | 0.914-3.349 | 0.091 | 0.307 | 0.033-2.890 | 0.302 | 0.818 | 0.221-3.031 | 0.764 |

**Additional file 2. Negative binomial regression analysis of entry, exit and feeding rate of *An. fluviatilis,* using positive control net as reference category**

*^@^ Reference category: Unwashed Duranet (Positive control)*
