# Supplementary material for: Evaluation of MAGNet, a long-lasting insecticidal mosquito net against Anopheles fluviatilis in experimental huts in India
Source: Malar J. 2019 Mar 6;18:59. doi: 10.1186/s12936-019-2692-3 (PMC6404338; doi:10.1186/s12936-019-2692-3)
Supplement: Supplementary file 3 — Additional file 3. Alpha-cypermethrin content in net samples before & after washing and after experimental hut trial. [file 12936_2019_2692_MOESM3_ESM.docx]

**Additional file 3. Alpha-cypermethrin content in net samples before & after washing and after experimental hut trial**

| **Treatment** | **AI content (g/kg)** | | | **AI retention(% of wash 0)** | **AI content (g/kg)**  **after hut trial** | **Compliance with specification** | **AI within-net variation (RSD)** |
| --- | --- | --- | --- | --- | --- | --- | --- |
|  | **Before washing** | **After 20 washes** | **After 25 washes** |  |  |  |  |
| Unwashed MAGNet | 5.8 | - | - | - | 5.2 | Yes | 1.7% |
| MAGNet washed 20 times | 5.6 | 5.3 | - | 95% | 5.0 | Yes | 3.2% |
| MAGNet washed 25 times | 5.7 | - | 5.1 | 90% | 4.9 | Yes | 3.3% |
| Unwashed Duranet | 7.1 | - | - | - | 5.2 | Yes | 2.2% |
| Duranet washed 20 times | 7.3 | 6.8 | - | 93% | 6.1 | Yes | 1.0% |
| Untreated net | <0.05 | - | - | - | <0.05 | Yes | 1.7% |
